# Integration of Social Media to Enhance Engagement With a Medical Education Website: A Content Analysis

**DOI:** 10.7759/cureus.104198

**Published:** 2026-02-24

**Authors:** Saveen K Sidhoo, Aaron Dou, Vaishvi Patel, Jessica Zhang, Daisy Sun, Paris-Ann Ingledew

**Affiliations:** 1 Radiation Oncology, University of Alberta, Edmonton, CAN; 2 Internal Medicine, University of Ottawa, Ottawa, CAN; 3 Medicine, McMaster University, Hamilton, CAN; 4 Radiation Oncology, British Columbia Cancer Agency, Vancouver, CAN

**Keywords:** oncology websites, online medical education, radiation and medical oncology, social media (sm), undergraduate and graduate medical education

## Abstract

Purpose: Social media (SM) use in medical education (MedEd) has increased, especially following the COVID-19 pandemic, as virtual platforms became essential for training. Social media offers unique tools to support diverse learning styles, address gaps in curricula, and promote digital literacy.

Objective: We examined how integrating social media with a medical education (MedEd) website enhanced engagement.

Methods: This is an original prospective content analysis. A review of literature identified best practices and interventions to improve the reach of SM platforms, such as quizzes and links to new content. Criteria to implement these interventions were based on the favorability of platforms from audiences, characteristics/features of various digital platforms, ease of content production, and engagement measurability. The number of likes, followers, and impressions, as well as the number and type of website users from X, Instagram, and the MedEd website, were collected between May 2022 and July 2025. Descriptive statistics, t-tests, and trend analysis were performed.

Results: During this period, 23 posts on X and 45 posts on Instagram led to follower increases of 53 to 112 (111.3%) and 170 to 449 (164.1%), respectively. Instagram posts averaged 13 likes and 473 impressions; X posts averaged three likes and 401 impressions. Website usage increased over time and averaged 544 users/month, with 337 new users/month, with 15,784/21,302 (74.1%) comprising healthcare professional students. Website traffic was significantly higher when social media posts were live (p=0.012).

Conclusions: Purposeful integration of social media for a MedEd website can expand reach and engagement of learners. This model serves as a proof of concept for other educators, and strategies are widely applicable.

## Introduction

Social media (SM) use has increased within education, allowing for the global transfer of knowledge at the touch of a button. While slow to adapt in healthcare, this trend has become more prevalent as a generation of physicians who grew up with SM begin to enter practice [1,2]. This was highlighted during the COVID-19 pandemic, when medical schools suddenly adopted a virtual learning platform to uphold training requirements while following social distancing practices [1,3].

Platforms such as X (formerly Twitter), Instagram, Spotify, and YouTube offer unique communication tools for various educational purposes, including mentorship, blended learning, promotion of student well-being, and professionalism [4,5]. SM also has many beneficial applications in medical education (MedEd). For example, studies have reported that the use of SM improved student communication and teamwork, where more students felt comfortable asking questions online than in person, and over 90% felt forums helped achieve their curricular learning objectives [6-8]. SM facilitates learning by reducing hierarchical paradigms, increasing curiosity for subject matters, and improving student performance on assessments [9]. Therefore, utilizing SM in MedEd represents an opportunity for educators to accommodate unique learning styles.

Many aspects of oncology care, including prospective career paths, remain underemphasized or absent in medical school curricula [10,11]. A Canada-wide survey found that 58% of educators and 67% of learners felt that undergraduate oncology education at their medical school was inadequate and the worst-taught medical subspecialty [12]. As such, minimal exposure or understanding of oncological issues and educational objectives translates to a lack of awareness of careers in cancer care. Indeed, many reports found that the majority of students had no exposure to Radiation Oncology (RO) and reported high levels of discomfort with oncological care [13-15]. These principles can also be extended to other oncology-related careers, as curricula and experience generally remain minimal. Consequently, initiatives are required to improve the supply-demand fundamentals for healthcare professionals trained in cancer care and are formally recommended by the European Society for Medical Oncology and American Society of Clinical Oncology [16]. Similar calls for action are also emphasized for Radiation Therapy, Medical Physics, and RO [16-18].

Learn Oncology (LO) is a free, online learning resource that aims to support medical students and healthcare providers to improve oncology education. The website (LearnOncology.ca) is currently used in more than 170 countries and attracts over 500 unique users per month, including healthcare professional students, resident physicians, practicing physicians, and other allied health professionals. The website consists of online learning modules, practice cases, whiteboard-style YouTube educational videos, and podcasts. In 2021, LO integrated SM platforms (Instagram and X) into its offerings with the goal of increasing the educational outreach of the website and providing additional learning modalities.

This study aims to evaluate the impact of the purposeful integration of SM to improve educational offerings and interaction of learners with a medical education website. The primary objectives were to assess the change in the number of users engaging (liking, commenting, viewing, following, etc.) with our SM platforms over time and to measure the change in website engagement in association with SM posts.

## Materials and methods

Study design

This study is an original prospective content analysis that examined the engagement of users on the LO website in correlation with the timing of SM posts on Instagram and X. A review of literature identified studies analyzing best practices in SM to engage learners for MedEd. A variety of interventions were identified to improve the reach of SM platforms: quizzes, links to literature, new online content, collaborative podcasts, “vlogs,” and artistic showcase pieces. Criteria to implement these interventions were based on the favorability of platforms from audiences, characteristics/features of various digital platforms, and ease of content production and engagement measurability.

Outcome measures

Analytics from Instagram and X platforms, as well as the LO website, were collected at the mid-point between the scheduled timing of each SM post. The number of likes, followers, accounts reached and engaged, and impressions (i.e., views) on Instagram and X were recorded from June 2022 to July 2025. The number of users and new users of the LO website were subsequently recorded at these respective points between May 2022 and July 2025.

Data analysis

Descriptive statistics were used to summarize characteristics of website users. An unpaired t-test was employed to compare the number of total LO website users at the halfway points between the times with and without SM posts. Results from the month of August were excluded from the final analysis to limit the effect of external confounding factors on the data. For example, an annual Canadian oncology initiative occurs at the end of August through the Canadian Association of Radiation Oncology in partnership with the Canadian Radiation Oncology Foundation, which was found to significantly increase website usage during this time. A trend analysis was also used to assess engagement over time across the three web platforms. All statistical analyses were performed using SPSS version 25 software (IBM Corp., Armonk, NY).

Ethics approval

Due to the nature of this study, REB/IRB approval and consent were not required.

## Results

At the start of the project, there were 79 posts on both X and Instagram. Since the initiation of the project, 23 additional posts were published on X, and 45 additional posts were published on Instagram. With these interventions, followers on Instagram increased by 164.1% from 170 to 449, and followers on X increased 111.3% from 53 to 112. On Instagram, the average number of likes per post was 13 (standard deviation (SD): 8, range: 3-44), and the average number of impressions (i.e., views) per post was 473 (SD: 494, range: 0-2,345). On X, the average number of likes per post was 3 (SD: 3, range: 0-10), and the average number of impressions per post was 401 (SD: 557, range: 43-2,225).

**Figure 1:**
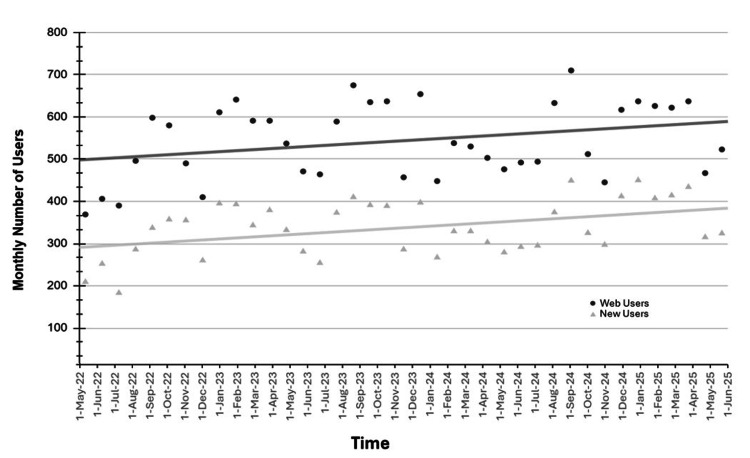
Monthly number of Learn Oncology website users (circle) and new website users (triangle) over time from May 2022 to July 2025

On average, 544 users (SD: 88, range: 369-710) engaged with the LO website per month between May 2022 and July 2025. Of these, 337 LO website users were new (SD: 65, range: 183-450) (Figure 1). Of the total website users, 8,658/21,302 (40.6%) were medical students, 4,210/21,302 (19.8%) were other allied healthcare providers, 2,784/21,302 (13.1%) were residents, 1,813/21,302 (8.5%) were nursing students, 1,308/21,302 (6.1%) were staff physicians, 1,032/21,302 (4.8%) were other students, 781/21,302 (3.7%) were radiation therapy students, and 716/21,302 (3.4%) were pharmacy students.

**Figure 2:**
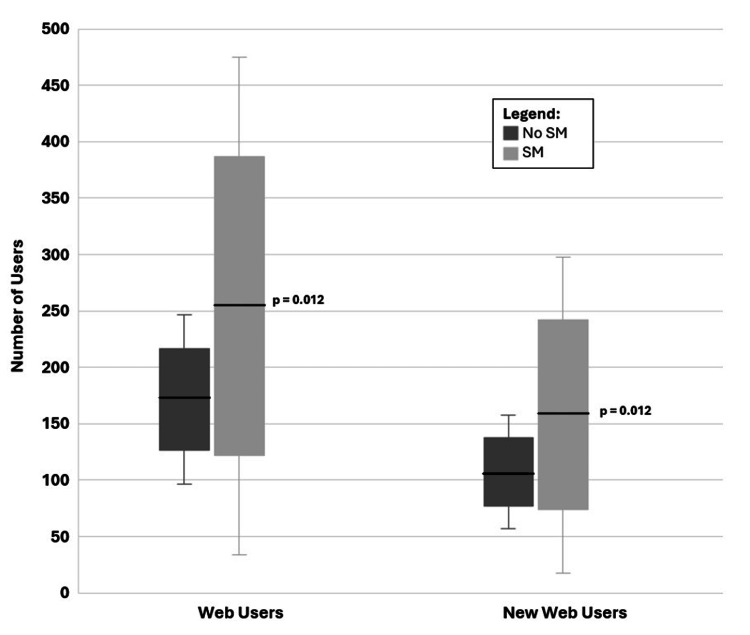
Average number of Learn Oncology website users (left) and new website users (right) without social media posts and with social media posts Box represents the standard error of the mean, and error bars represent the standard deviation (p=0.012).

The mean number of total and new LO website users during the times at which SM posts were live were 254 users (standard error mean (SEM): 44, SD: 221) and 158 users (SEM: 28, SD: 140), respectively (Figure 2). Conversely, the average number of LO website users and the mean number of new LO website users during the times at which no SM posts were live were 172 users (SEM: 15, SD: 75) and 107 users (SEM: 10, SD: 50), respectively (Figure 2). Independent sample t-test scores were calculated to be -1.177 (F: 6.758, df: 48, p=0.012) and -1.697 (F: 6.878, df: 48, p=0.012) for total LO website users and new LO website users, respectively.

## Discussion

In this study, we demonstrate purposeful integration of SM and the downstream effects on digital media literacy competencies. We found that the use of Instagram and X was associated with an increase in the number of followers and engagers with oncology MedEd content over time. Simultaneously, there was a trend of increased user engagement with the LO website over time (Figure 1). There was a significant increase in LO website users (Figure 2), most of whom were new website users when SM posts went live. These results show that the purposeful integration of SM techniques is associated with an increase in the educational outreach of a MedEd website to learners. This supports previous findings of (a) the widespread use of SM by undergraduate students and (b) students favoring SM interventions when incorporated into their studies [1]. By extension, these findings suggest that intentional SM strategies may also support improvements in clinical knowledge, skills, and early career exploration by broadening access to educational materials [19].

Further subanalysis found that SM posts that garnered the most engagement were those made in collaboration with other individuals, schools, or initiatives, such as podcasts. For example, a short series of podcasts that were employed, “Cancer Careers,” explored various perspectives of individuals sharing their career paths within oncological specialties. We found that collaboration with individuals who openly shared their journeys was associated with an increase in engagement with SM posts, likely due to the featured guests sharing and broadening the outreach of SM posts with our target audience. Podcasts are also an effective supplemental learning tool for the fundamentals of oncology for physician residents, with increased retention of knowledge and educational value beyond clinical rotations [20].

The aim of LO is to provide learning support for medical students and healthcare professionals with oncology education. Within Canada, cancer has now surpassed cardiac disease as the leading cause of mortality, and its prevalence continues to grow as 40% of Canadians develop cancer in their lifetime [21]. Most healthcare providers are likely to be involved at some point in cancer care, thereby highlighting the need for awareness of potential complications associated with various malignancies, as well as approaches in managing oncology-related issues (i.e., screening, diagnosis, treatment, and/or surveillance).

As such, digital media literacy in healthcare is critical when it comes to accessing, analyzing, and producing medical information. This literacy is defined by the components of content, competencies, and the interrelations between the two [10].

Content for SM literacy involves developing awareness, understanding, and knowledge across three dimensions: the self, the medium, and reality. The “self” refers to users’ choices, motivations, networks, and digital spaces they interact with as they construct and analyze online information. The “medium” captures how various platforms shape the communication of knowledge based on their characteristics and features. For example, use of X is for “information and informality,” and Instagram for “stylized self-presentation with multifaceted communication spaces, both intimate and semi-public” [22]. “Reality” links digital content to real-world meaning, which connects the efficacy of digital literacy education programs to the media produced. Within our study, these dimensions were demonstrated through the production, engagement, and dissemination of LO material among engagers.

Competencies refer to the skills needed to analyze, evaluate, and produce SM content [10]. This involves individuals monitoring content and network choices, engagement behaviors, and identifying the beliefs, values, and experiences behind the messages. By assessing the realism of content, users then develop and disseminate messages for both personal and collective goals. In this study, growing and sustained activity on SM platforms and the LO website suggests that learners were developing these competencies. This indicates that LO may assist educators and support learners in building the skills required for safe and effective oncological care for patients.

Consequently, the ideal target audience for such initiatives are students, who are still exploring career options. A single institutional study showed that incorporating virtual electives into their curriculum resulted in students reporting higher understanding and interest in oncological careers [23]. In our study, a large proportion of LO users were students, suggesting strong relevance for this group. As such, integrating SM with medical education platforms like LO may play a meaningful role in addressing gaps within oncology education and the broader workforce pipeline. With LO’s whiteboard videos, tumor site-based summary notes, podcasts, quizzes, and virtual workshops, we aim to improve students’ comfort levels in oncology topics, which may translate into stronger clinical skills, increased interest in oncology-based careers, and improved accessibility to mentorship. Furthermore, LO’s materials were reviewed by experts in the field and updated frequently with practice-changing guidelines, and are available online for free, making them easily accessible globally. Through our iterative integration of key interventions, this study demonstrated a progressive and expanded reach of the MedEd site. Taken altogether with the results of previous literature, we were able to synthesize possible reasons that students may benefit from the incorporation of SM (Table 1), and the lessons here may apply to a range of medical educators.

**Table 1:** Synthesized reasons for students benefitting from the incorporation of SM into the education curriculum based on study results and previous literature findings SM: social media

Factor	Reasons
Accessibility	Many individuals use SM; user-friendly; distant learning opportunities, broadcasting; transfer of knowledge with others is easier; visual and/or audio modes; personalized learning experience; cost-effective
Communication and collaboration	Decreased sense of loss; easier to allow “quieter” individuals to “speak up” in forums compared to in-person group settings; facilitates teamwork, reduces potential for hierarchies/power dynamics; allows for real-time feedback
Flexibility	Ability to access sources at any time of the day; can adjust the speed of videos, thereby improving time management and multitasking
Professionalism	Increased mentorship/networking opportunities; increased awareness of new subjects and careers; improved awareness of professional practices/behaviors on SM
Scholar	Increased engagement, enjoyment, and curiosity; increased work efficiency; improved scores on assessments; improved critical thinking skills; wider range of educational content/resources

Our results, however, must be interpreted within the context of some limitations. The Modern Kirkpatrick Model of Training provides a framework that organizations can use to gather data and identify the impact that certain programs or initiatives may have on the organization [24]. As per this model, there are four levels of evaluation that could occur in MedEd: (1) the reactions from students toward an intervention, (2) the resulted learning and increased knowledge students develop from the intervention, (3) the behavioral change and improvement in students after applying skills in a clinical setting, and (4) the effects students’ performances have in clinical flow. The design of this study did not allow for the evaluation of the changes in knowledge retention, behavioral changes, and effects of performance that our interventions may have had on our target audience. Rather, we sought to determine the reaction learners had to our various interventions, as well as the number of users engaged over time. Additionally, this was a non-randomized study, which is susceptible to selection bias and confounding variables. There is the inability to prove that website traffic spikes were directly caused by social media posts or merely correlated. In order to minimize this, identified outliers were excluded from the final analysis, which may have impacted significance.

## Conclusions

Overall, the incorporation of SM into MedEd continues to evolve. Here, we discuss key digital media literacy principles to incorporate SM into a MedEd website. With the integration of these principles, we demonstrate a progressive and expanded reach of this MedEd site. The lessons here may be applied to a range of medical educators as they develop new online learning tools and in addressing gaps and workforce shortages in oncology-related careers. Future work could evaluate the extent to which SM-based engagement translates into measurable knowledge acquisition, skill development, and educational benefits for learners.
